# Histamine Dynamics During Ingestive Behavior Measured by the Novel Biosensor HisLightG

**DOI:** 10.1111/jnc.70142

**Published:** 2025-07-03

**Authors:** K. L. Volcko, A. Gresch, B. Benowitz, H. Taghipourbibalan, M. Visser, V. Rohner, G. D. Stuber, A. G. Gordon‐Fennell, T. Patriarchi, J. E. McCutcheon

**Affiliations:** ^1^ Department of Psychology UiT The Arctic University of Norway Tromsø Norway; ^2^ Institute of Pharmacology and Toxicology University of Zürich Zürich Switzerland; ^3^ Center for the Neurobiology of Addiction, Pain, and Emotion, Department of Anesthesiology and Pain Medicine, Department of Pharmacology University of Washington Seattle Washington USA; ^4^ Neuroscience Center Zürich University and ETH Zürich Zürich Switzerland

**Keywords:** appetite, biosensors, feeding, photometry

## Abstract

The neurotransmitter histamine is involved in control of food intake, yet its dynamics during individual feeding episodes remain unexplored. Therefore, we used the novel genetically‐encoded histamine sensor, HisLightG, combined with fiber photometry to measure histamine release in two hypothalamic regions critical for the food‐suppressive effects of histamine, the paraventricular nucleus of the hypothalamus (PVH), and the ventromedial hypothalamus (VMH). Male mice were tested under different conditions to assess whether hunger, time of day, or the caloric content of the solution they were given affected histamine fluctuations. We found that histamine levels changed rapidly in response to eating. These histamine fluctuations were influenced by experimental conditions, with slightly smaller responses when the test solution was sucralose (both regions) or during the light cycle (PVH only). Notable regional differences were identified, such that in the PVH histamine rebounded to above baseline levels, whereas in the VMH histamine remained lower than baseline for at least 10 s after licking ceased. In a separate cohort of male and female mice, enhancing histamine tone via administration of a histamine precursor (L‐histidine) reduced the number of licks across multiple sucrose concentrations. Together, these findings indicate that histaminergic activity is modulated rapidly during ingestive episodes, and that understanding these release patterns will give insight into histamine's role in appetite suppression.

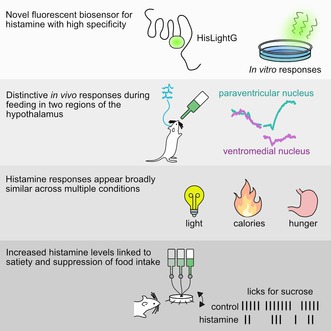

## Graphical abstract

Here, Volcko et al. describe a novel fluorescent sensor that allows changes in the molecule histamine to be recorded in the brain. They first show that the sensor is specific for histamine when used in vitro in cell culture. Then, they use fiber photometry to measure histamine release in two different regions of the brain while mice are drinking a nutritive solution. They find that the two brain regions respond quite differently. These responses seem broadly insensitive to changing conditions (e.g., lighting, caloric content of the food, and hunger). Finally, they show that increasing histamine levels in the brain suppress feeding, suggesting a link between histamine and satiety.

**Abbreviations**
H1R: H1 receptor
PVH: paraventricular nucleus of the hypothalamus
RRID: Research Resource Identifier (see scicrunch.org)
VMH: ventromedial hypothalamus

## 1 Introduction

The mechanisms that govern meal termination and reductions in food intake are complex and intertwined. The neurotransmitter and neuromodulator histamine plays an important role in ingestive behavior. Many well‐known satiety signals, including leptin (Morimoto et al. 1999; Toftegaard et al. 2003), amylin (Lutz et al. 1996; Mollet et al. 2003), cholecystokinin (Attoub et al. 2001), and glucagon‐like peptide‐1 (Gotoh et al. 2005), have been shown via pharmacological and genetic methods to require histamine in order to exert their full effects on eating behavior, though the degree to which these signals rely on histamine varies. Histamine may be responding directly to some of these signals—for example, amylin fibers and histaminergic neurons are found in close proximity (D'Este et al. 2001)—but there is likely also indirect input, such as through the melanocortin system (Michael et al. 2020) or noradrenaline (Kurose and Terashima 1999).

Histamine is involved in many physiological processes besides eating, and can either stimulate or suppress food intake depending on the precise location and receptor subtype activated. The bulk of previous research measuring histamine levels in the brain used microdialysis, a technique which is limited by a relatively large sampling area (e.g., “anterior hypothalamus”) as well as low temporal resolution (e.g., 20–30 min between samples). Fast‐scan cyclic voltammetry has also been used to measure histamine with high temporal resolution in vivo (Berger et al. 2022; Samaranayake et al. 2015). However, properties of the molecule and its release patterns make these studies difficult to perform and have limited utility for studying spontaneous histamine release during naturally occurring behaviors (i.e., outside of stimulation of histamine containing fibers). Recent advances in genetically‐encoded fluorescent neurotransmitter sensors have allowed in vivo neurotransmitter release to be combined with fiber photometry yielding measurements with high chemical, temporal, and spatial precision (Patriarchi et al. 2019; Simpson et al. 2024). So far, a pair of histamine sensors, named GRAB‐HA1m and GRAB‐HA1h, have been developed for in vivo use (Dong et al. 2023). Previous studies using these sensors showed histamine fluctuations during sleep‐wakefulness (Dong et al. 2023), after an aversive stimulus (Lin et al. 2023), and, of particular interest to us, in the medial septum during eating after 24 h fasting (Xu et al. 2022). To our knowledge, however, no one has previously recorded histamine fluctuations in the hypothalamus using fiber photometry during eating behavior under a variety of hunger states. Moreover, unlike the other studies cited, we use the novel histamine sensor HisLightG (Kagiampaki et al. 2023) in this work. We also provide a direct side by side comparison between the properties of our sensor and those of the previously available ones, highlighting key differences between the tools.

Central histamine‐producing neurons are located in the tuberomammillary nucleus in the posterior hypothalamus (Panula et al. 1984; Watanabe et al. 1984), and project widely throughout the brain, including many hypothalamic nuclei (Lin et al. 2023; Panula et al. 1989). Histamine acts on several receptor subtypes, although it is the H1 receptor (H1R) that mediates suppressive effects of histamine on food intake (Fukagawa et al. 1989; Kurose and Terashima 1999; Lecklin et al. 1998; Masaki et al. 2004; Morimoto et al. 1999; Ookuma et al. 1989; Sakata et al. 1988). H1Rs are expressed in many parts of the brain, with a high density found in both the ventromedial hypothalamus (VMH) and the paraventricular nucleus of the hypothalamus (PVH) (Futagawa et al. 2024). Infusions of H1R antagonists, or drugs to inhibit local histamine production, into specific hypothalamic nuclei have indicated that these two regions—the VMH and the PVH—are the critical sites of action for histamine to attenuate eating (Ookuma et al. 1989, 1993; Sakata et al. 1988, 1990, 1991).

We wanted to examine the patterns of histamine release in the PVH and the VMH during eating bouts in mice, using fiber photometry and the new histamine sensor, HisLightG (Kagiampaki et al. 2023). Histamine levels in the hypothalamus are sensitive to the time of day (Doi et al. 1994; Mochizuki et al. 1992; Sakata et al. 1988). We therefore recorded histamine fluctuations during both the dark and light phases. Additionally, histamine levels may or may not be influenced by hunger status (Itoh et al. 1991; Sakata et al. 1994). Consequently, we measured histamine in both ad libitum and food‐restricted conditions. Finally, in a separate experiment to determine how histamine levels shape consumption over a range of rewarding solutions, we manipulated histamine levels by providing the precursor, L‐histidine, and measured licking for different concentrations of sucrose.

## 2 Methods

### 2.1 In Vitro Experiments

#### 2.1.1 Cell Culture, Confocal Imaging, and Quantification

For this study HEK293T cells from (ATCC cat. no CRL‐3216, cell line last authenticated by the provider) which have been passaged up to 32 times (P32) were used. This cell line has not been listed as a commonly misidentified cell line by the International Cell Line Authentication Committee (ICLAC, version 13). The cells were cultured in DMEM medium (Thermo Fisher cat. no 41966029) supplemented with 10% FBS (Thermo Fisher cat. no A5256701) and 100 μg/mL Penicillin–Streptomycin mix (Thermo Fisher cat. no 15240062) and incubated at 37°C with 5% CO_2_. Cells were transfected at 50%–60% confluency in glass‐bottomed dishes using PolyFect Transfection Reagent (Qiagen cat. no 301105) according to manufacturer instructions, and used for follow‐up experiments 24–48 h after transfection. Before imaging, cells were rinsed with 1 mL of Hank's Balanced Salt Solution (HBSS, Thermo Fisher cat. no 14025100) containing CaCl_2_ and MgCl_2_ supplemented with 30 mM HEPES (Thermo Fisher cat no. 15630080). All cells were imaged at room temperature in glass bottom dishes using HBSS with 30 mM HEPES. Imaging was performed using ZEN Blue software on an inverted Zeiss LSM 800 confocal microscope equipped with 488 nm laser and a 40X oil‐based objective. During timelapse imaging, ligands were applied manually at 10‐fold the final concentration (1:9 dilution of ligand solution to buffer volume on the cells) using a micropipette. For quantification of the fluorescence response ∆F/F_0_, regions of interest (ROI) enclosing isolated cell membranes were selected manually using the threshold function of Fiji (ImageJ). Sensor response (∆F/F_0_) was calculated as follows: (F(t)−F_0_)/F_0_ with F(t) being the ROI mean gray value at each time point (t), and F_0_ being the mean gray value of the 10 timepoints immediately prior to ligand addition. For the quantification of the basal brightness, we measured the mean gray value of one ROI per image (179.44 μm by 179.44 μm) which was composed of the 10% brightest pixels in the image (corresponding to the plasma membranes of the expressing cells) selected using the threshold function in Fiji (ImageJ). For each of the three indicators a total of *n* = 6 images were taken (*n* = 2 images on 3 days).

### 2.2 Fiber Photometry Experiments

#### 2.2.1 Animals

Adult male C57BL/6NRj mice (RRID:IMSR_RJ:C57BL‐6NRJ; 6–8 weeks old) were purchased from Janvier (France) and housed in a temperature‐ and humidity‐controlled room, maintained on a 12:12 light: dark cycle (lights‐on at 00:00). Two mice shared each cage, separated by a perforated divider that allowed visual, olfactory, and auditory communication but prevented major physical contact. Water and food were available ad libitum unless mice were undergoing food restriction. All animal care and experimentation followed the EU directive 2010/63/EU for animal experiments and was approved by the National Animal Research Authority in Norway (FOTS protocol 22315).

#### 2.2.2 Surgical Procedures

Surgery was performed after a minimum of 5 days of habituation to the animal facility. Mice (*n* = 24; one mouse died from surgical complications) were anesthetized with 1.5%–2% isoflurane suspended in air, and secured in a stereotaxic apparatus (Kopf, Tujunga, CA, USA). Each mouse was given subcutaneous injections of buprenorphine (0.1 mg/kg; Temgesic) and meloxicam (5 mg/kg; Metacam) to provide systemic analgesia, as well as bupivacaine (1 mg/kg; Marcain) as a local anesthetic. A small burr hole was made above the target area (see Table 1 for coordinates; all coordinates based on a flat skull) and 500 nL (0.6 × 10^9^ vg) of AAV‐9/2‐hSyn1‐chI‐HisLightG‐WPRE‐bGHp(A) (#10264; ETH Viral Vector Facility) was infused at a rate of 100 nL/minute using a Nanofil syringe with 33 g blunt needle and UMP3 microsyringe pump (WPI; Sarasota, FL, USA). The needle remained in place for 10 min to allow the virus to diffuse. Next, a fiber optic cannula (1.25 mm ceramic ferrule, 0.4 NA, 400 μm core; RWD) was lowered slowly to just above the virus injection. The fiber optic was secured to the skull with dental cement (Superbond C&B). Each mouse received meloxicam 2 days postoperatively.

**TABLE 1:** Surgical coordinates.

Target	Medial–lateral	Anterior–posterior	Dorsal–ventral (virus)	Dorsal–ventral (fiber)
Paraventricular nucleus of the hypothalamus	0.3 mm lateral to midline	0.8 mm posterior to bregma	4.70 mm ventral to skull	4.60 mm ventral to skull
Ventromedial nucleus of the hypothalamus	0.3 mm lateral to midline	1.20 mm posterior to bregma	5.65 mm ventral to skull	5.55 mm ventral to skull

*Note:* Coordinates used to target the PVH and VMH are provided, including the medial–lateral, anterior–posterior, and dorsal–ventral coordinates relative to bregma and based on a flat skull.

#### 2.2.3 Testing Apparatus

Testing took place in operant chambers (24 cm × 20 cm; Med Associates, Fairfax, VT). The fan was on continuously during testing. When mice were tested during their dark phase, the house light was off in the testing chambers, and when the mice were tested during their light phase, the house light was on in the operant chambers. Solutions were delivered through blunted 18 g needles attached to 5 mL syringes via flexible tubing. These syringes were mounted on syringe pumps that dispensed the solution when licks were detected via a contact lickometer. These pumps were controlled by MEDPC‐V software (Med Associates).

While the mice freely explored the operant chambers and licked solutions, histamine activity was recorded via fiber photometry. An RZ10x system and Synapse software (Tucker‐Davis Technologies, Alachua, FL, USA) controlled two LED light sources, one blue (465 nm) and one violet (405 nm). The blue light was sinusoidally modulated at 330 Hz and the violet at 210 Hz. A fluorescence minicube (Doric Lenses, Quebec, Canada) combined the blue and violet wavelengths. A 400 μm optical patch cord (Doric Lenses) transmitted the light to the mouse, where the cable and ferrule were attached by a black ceramic sleeve (RWD). This same cable was used to collect light emitted from the brains of the mice. This light was detected by a femtowatt photoreceiver (#2151; Newport, Irvine, CA, USA).

#### 2.2.4 Testing Procedures

Each mouse underwent a series of testing sessions in different blocks, with each block a different condition. The order of the blocks was the same for all mice. Testing began at least 12 days after surgery and after mice had been habituated to having a cable attached to their fiber optic implant. The four blocks were (1) ad libitum light, (2) food restricted dark, (3) ad libitum dark, and (4) ad libitum sucralose. In the ad libitum light block (“Light”), mice with ad libitum food and water were tested during the light phase. They were placed in operant chambers for three 30‐min sessions on different days, in which 1 kcal/mL “Ensure” (Nestle Resource Complete neutral flavor) was continuously available through a spout. In the food restricted dark block (“Restricted”), mice were tested in the dark while food restricted. Food restriction was achieved by giving 2.5–3 g of chow to each mouse each day; this resulted in approximately 5%–10% weight loss. In this phase they were again given “Ensure” during three 30 min sessions. The ad libitum dark block (“Dark”) was identical to the Light block except mice were tested during their dark phase and only tested in two sessions. Lastly, in the ad libitum sucralose block (“Sucralose”) mice were tested without food restriction in the dark, but given 2 mM sucralose (Merck cat. no 69293) in the single 30 min session.

#### 2.2.5 Histology

Only mice with virus and fibers placed correctly were included in the final analysis. To check placements, brains were collected at the end of the experiment. Briefly, mice were deeply anesthetized with 0.3 mL of ZRF mix (zolazepam, 3.3 mg/mL; tiletamine, 3.3 mg/mL; xylazine, 0.45 mg/mL; fentanyl, 2.6 ug/mL). The mice were then transcardially perfused with heparinized saline followed by 4% paraformaldehyde. Brains were removed, postfixed overnight in 4% paraformaldehyde, and placed in 30% sucrose with Proclin 150 (Sigma cat. no 49376‐U). A microtome (Leica, Deer Park, IL; USA) was used to cut brains into 40 μm coronal sections. Every third section was directly mounted onto a Superfrost Plus slide and coverslipped. The native fluorescence of the viral injections was used to determine the location and spread of the injection and the fiber tract used to assess the location of the implant. Four out of 11 mice with PVH placement were excluded, and five out of 12 mice with VMH placements were excluded due to poor viral expression or fiber placements. This resulted in a group size of *n* = 7 per region.

### 2.3 Histamine Manipulation Experiments

#### 2.3.1 Animals

Adult male and female C57BL/6J mice (*n* = 4 of each sex) were bred and housed in the animal facility at University of Washington. Mice were kept on reverse 12 h light: dark cycle (lights‐on at 21:00) and behavioral experiments were conducted within the dark cycle. Mice were single‐housed to prevent damage to the head fixation device, provided with water ad libitum, and during experiments were food restricted to ~90% of free‐feeding weight by providing a ration of food (2.5–4 g/day) depending on weight change from the previous day. All animal procedures were pre‐approved by the Institutional Animal Care and Use Committee (IACUC) at University of Washington (protocol 4450‐01).

#### 2.3.2 Surgery

Mice (*n* = 8) were anesthetized with isoflurane (5% for induction, 1.5%–2% for maintenance suspended in O_2_), injected with analgesic (carprofen, 10 mg/kg, s.c.), and the scalp was shaved using electric clippers. Mice were mounted in a stereotaxic frame with heat support, and the scalp was injected with local anesthetic (lidocaine, 2%, s.c.) and sterilized using ethanol and betadine. An incision was made using a scalpel, and the skull was cleared of tissue and scored with the pointed end of the scalpel. The skull was leveled, and two micro screws were inserted into burr holes drilled in the lateral portion of the occipital bone, with one additional screw placed in the rostral portion of the skull. A head fixation device (a head plate; 0.8 mm stainless steel 304 series, sendcutsend.com) was attached to the skull and screws using Super Glue, and then dental cement was used to encase a portion of the head bar and the screws. Once fully set, mice were removed from the stereotaxic frame, allowed to recover with heat support, and then returned to their home cage and allowed to recover for at least 1 week before food restriction began. Mice were monitored daily for 3 days following surgery for signs of postoperative pain or distress, including hunching, piloerection, or lethargy. If any of these signs were observed, veterinary staff were notified, and an additional subcutaneous dose of carprofen (10 mg/kg) was administered. However, no mice used in this study exhibited overt signs of distress requiring additional analgesia.

#### 2.3.3 Behavior in Multispout Apparatus

Mice were assessed daily for signs of distress prior to each head‐fixation session. Indicators of distress included unexpected weight loss (defined as < 85% of presurgical body weight), hunching, piloerection, lethargy, or reduced activity. To minimize stress associated with head‐fixation, mice underwent progressive habituation over several days as described in (Gordon‐Fennell et al. 2023). Briefly, these steps were as follows: (1) transport to the behavioral suite and handling by the experimenter, (2) scruffing, handling by their head bar, and exposure to the fixation tube (a 50 mL conical tube), (3) scruffing and brief placement in the tube, and (4) head‐fixation with free‐access lick training with 30% sucrose for 10 min. During each head‐fixation session, mice were monitored for signs of acute distress, including continuous vocalizations or struggling within the tube. Animals exhibiting these signs were removed from the study. No mice in this study exhibited behaviors that warranted removal. Following this habituation, mice were then trained on the multispout apparatus with multiple concentrations of sucrose available. The assay consisted of daily sessions with 100 trials with 3 s access to one of five different sucrose concentrations (0% (water), 5%, 10%, 20%, and 30%, 20 trials of each). Solutions were presented pseudorandomly with an inter‐trial interval of 12.5 ± 2.5 s. Licks were detected using a capacitive touch sensor (Adafruit MPR121). Once mice had reached stable behavior in which lick rate showed a monotonic relationship with sucrose concentration, the pharmacological manipulation began. In two consecutive sessions mice received either L‐histidine (500 mg/kg; Sigma‐Aldrich cat. no 53340) or vehicle (saline) i.p. 2 h before being tested in the multispout apparatus. Order of administration was counterbalanced across mice and sexes.

### 2.4 Experimental Design and Statistical Analysis

For all experiments, data were processed with Python and statistical analyses were conducted using JASP (0.19.3; https://jasp‐stats.org/). Sample size justification for all experiments is provided in Appendix S1.

#### 2.4.1 In Vitro Experiments

Outliers (2 standard deviations above or below the group mean) were removed. Out of an initial *n* = 30 cells per neurotransmitter, this resulted in *n* = 28 for histamine, HBSS, acetylcholine, and serotonin, *n* = 29 for glutamate and norepinephrine, and *n* = 30 for dopamine and GABA. Data were then assessed for normality using the Shapiro–Wilk test. Finally, potential differences in fluorescence relative to the control, HBSS, were determined by Welch ANOVA with Dunnett's post hoc test. All experiments were repeated 3 times with similar results. A *p* value of < 0.05 was considered significant.

#### 2.4.2 Fiber Photometry Experiments

Data from mice in the PVH cohort and the VMH cohort were analyzed separately, except for when they were directly compared and when they were combined. Post hoc tests were Holm corrected for multiple comparisons. TDT files included timestamps of licks relayed to the TDT system through MED‐PC and its lickometer, as well as the photometry streams for HisLightG and the 405 nm isosbestic control. The photometry signal was corrected for bleaching and artifacts by using FFT‐based correction method to subtract the 405 nm signal from the 465 nm signal, as previously described (Konanur et al. 2020). No blinding was performed.

Several aspects of licking behavior were analyzed: average licks per session, average number of bouts per session, and average length of bouts per session. Lick bouts were defined as a cluster of 3 or more licks with at least 10 s separating one bout from another. This method of defining clusters of licks was used to remain consistent with those used in the photometry and is not the standard microstructural definition. Repeated measures ANOVA with Condition (Restricted, Light, Dark, and Sucralose) as a within‐subjects factor was conducted for each of these parameters. We did not expect mice with PVH or VMH placements to differ in behavior. This was confirmed in a preliminary analysis finding no main effect of Region in licks, number of lick bouts, or length of lick bouts. We therefore combined mice from both regions for subsequent analyses. Normality of data was assessed through examining the Q‐Q plots of residuals, and sphericity was tested and Greenhouse–Geisser corrected when this assumption was violated.

Peri‐event traces (hereafter, “snips”) of the photometry trace corresponding to the 5 s before a lick bout, and lasting 10 s after licking ended, were created for each lick bout for each mouse. Of the total lick bouts, we created snips for those lasting at least 8 s (in the Restricted analysis of PVH vs. VMH) or 4 s (for the analysis between conditions, due to the low number of longer lick bouts in the Sucralose condition). Artifacts were removed by comparing the absolute difference between consecutive time points, and if this was greater than a threshold of 12, that lick bout was discarded. To account for different durations of each bout, only the first 6 s and last 2 s of licking were included. Snips were normalized by z‐scoring, using the 5 s before licking as baseline; a baseline was calculated for each snip.

We used a strategy similar to that described in (Jean‐Richard‐dit‐Bressel et al. 2020) to conduct waveform analyses of histamine fluctuations. First, we created a bootstrapped estimate of the mean using all lick bouts from a given region and condition, sampling 1000 times with replacement. A 95% confidence interval was then constructed around the mean. We set a consecutive threshold of six bins (equivalent to 0.6 s), corresponding to the low‐pass filter of the photometry signal. As such, if the confidence interval did not include zero for at least six consecutive time bins, the period was determined as a true fluctuation in histamine. Similarly, for a comparison between PVH and VMH in the restricted condition, we compared the confidence intervals for the PVH and VMH and when they were not overlapping for at least six consecutive time bins, we considered them different from each other.

#### 2.4.3 Multispout Experiments

For the multispout experiments, we analyzed the data with repeated‐measures ANOVA with sucrose concentration and injection (vehicle or L‐histidine) as within‐subjects factors. Normality of data was assessed through examining the Q‐Q plots of residuals, and sphericity was tested and Greenhouse–Geisser corrected when this assumption was violated. We also performed planned comparisons between injections at each sucrose concentration to test the hypothesis that at each concentration, mice would lick less when given L‐histidine injections than vehicle injections.

## 3 Results

### 3.1 In Vitro Results

**FIGURE 1:**
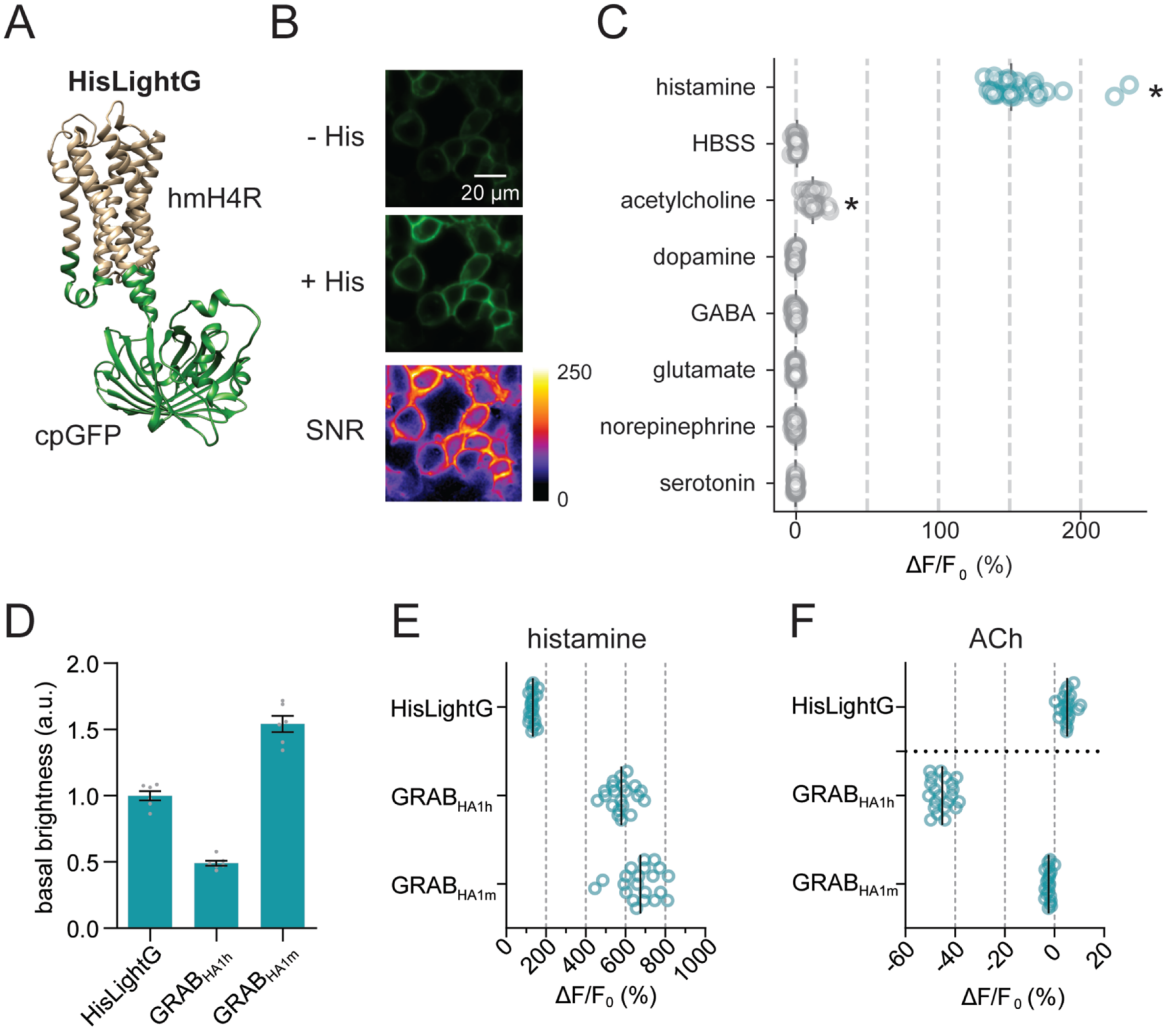
In vitro results. (A) Structural model of HisLightG (Kagiampaki et al. 2023) obtained through AlphaFold2 (Mirdita et al. 2022). Indicated in green are the grafted components from dLight1.3b, and in tan the original sequence from the human H4 histamine receptor. (B) Representative images of HisLightG‐expressing HEK293T cells in the presence or absence of histamine (10 μM) and corresponding signal‐to‐noise (SNR) heatmap. (C) Quantification of the fluorescence response of HisLightG to different non‐ligand neurotransmitters (10 μM) in HEK293T cells. Data are shown as response of individual cells, with vertical lines indicating the mean for that neurotransmitter. *p < 0.05 relative to HBSS. (D) Quantification of the basal brightness of HisLightG, GRAB‐HA1h and GRAB‐HA1m measured in HEK293T cells. Data are shown as mean fluorescence brightness of thresholded plasma membranes normalized to the basal brightness of HisLightG with error bars as SEM. (E) Quantification of the fluorescence response of HisLightG, GRAB‐HA1h and GRAB‐HA1m to histamine (10 μM) in HEK293T cells. Data are shown as response of individual cells, with vertical lines indicating the mean of all cells. (F) Quantification of the fluorescence response of HisLightG, GRAB‐HA1h and GRAB‐HA1m to acetylcholine (10 μM) in HEK293T cells. Data are shown as response of individual cells, with vertical lines indicating the mean of all cells.

The fluorescent sensor HisLightG was created by combining the human H4 histamine receptor with a portion of the fluorescent sensor for dopamine, dLight1.3b (Figure 1A; Kagiampaki et al. 2023). To confirm the specificity of HisLightG to histamine, we performed in vitro tests in which HEK293T cells were transfected and the fluorescent response to a variety of neurotransmitters was measured. Relative to control (Hank's Buffered Saline Solution), addition of 10 μM histamine caused an approximate 150% increase in sensor fluorescence (Figure 1B; *p* < 0.001). Application of acetylcholine (10 μM) also led to a very small but significant increase in fluorescence (*p* < 0.001). This response was only a 12% increase, approximately 13 times smaller than the response to histamine, and it is unlikely to substantially contaminate fluorescent signals measured in vivo. All other neurotransmitters tested were without effect (Figure 1C; all *p*'s > 0.05). Next, we compared the basal brightness (Figure 1D) and fluorescence response (Figure 1E) of HisLightG to that of the previously published GRAB‐HA1h and GRAB‐HA1m (Dong et al. 2023). The basal brightness of HisLightG is intermediate compared to the two other indicators, while its dynamic range is lower (GRAB‐HA1h ~580%, GRAB‐HA1m ~680%). Because we observed a response of HisLightG to acetylcholine and the GRAB indicators are based on the same receptor (human H_4_R) we tested the fluorescent response of all three indicators to the application of acetylcholine (at 10 μM final concentration applied as a 1:9 dilution of a 100 μM stock concentration) side‐by‐side. While acetylcholine only caused minimal fluorescence changes at HisLightG and GRAB‐HA1m, we measured a fluorescence decrease of approximately 45% for GRAB‐HA1h (Figure 1F).

### 3.2 Photometry Results

#### 3.2.1 Licking Behavior Across Time of Day, Hunger State, and Caloric Content

**FIGURE 2:**
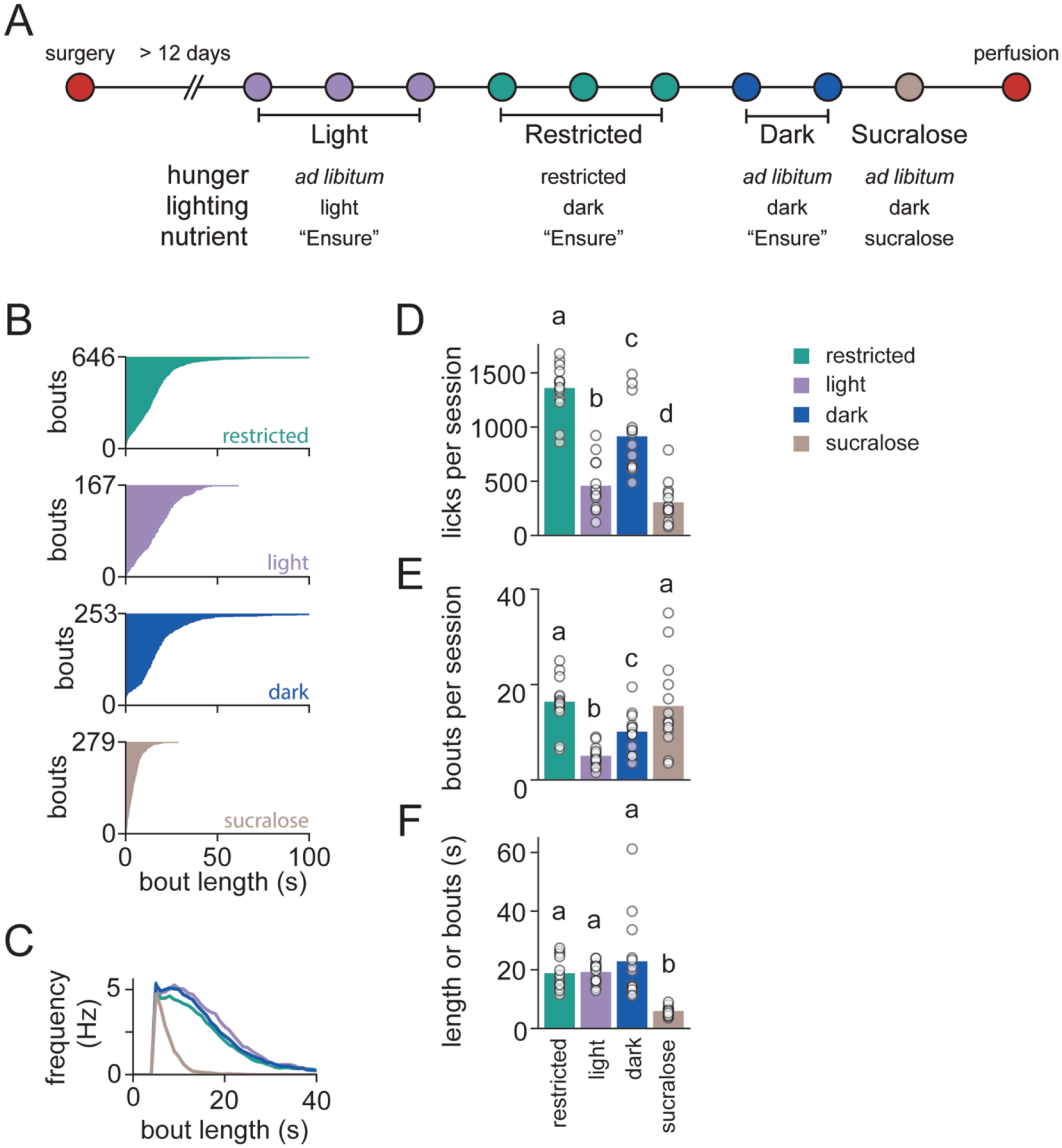
Schematic and licking behavior. A timeline of the experiment shows the number and order of photometry sessions; mice in both the PVH and VMH cohorts were tested in the same conditions and order of conditions (A). The total number of lick bouts (i.e., those that contained at least three licks and which were separated from another licking bout by at least 10 s) across all mice and sessions for each condition, plotted against bout length, is displayed (B). Lick frequency was similar in all conditions except Sucralose (C). Condition affected how much mice licked on average in each session (D). Condition also influenced the number of bouts per session (E). The average length of bouts, in seconds, also differed by Condition (F). Bars are means and circles individual data points, lower‐case letters indicate *p* < 0.05 (e.g., a is different from b).

Licking behavior in the different conditions—Restricted, Light, Dark, and Sucralose—was compared across all mice that were included in the photometry analysis, without regard to whether the PVH or VMH was targeted. The schematic in Figure 2A details how hunger state, time of day, and nutritional value of the solution were manipulated in each of these four conditions. We plotted total lick bouts of any length (the number of bouts that included at least three licks which were separated by another licking bout by at least 10 s, with no criteria for length in time of the lick bouts) in all sessions against bout length, for each of the conditions (Figure 2B) as well as lick frequency for each condition (Figure 2C). Next, we divided these data by session and statistically examined average licks per session, average number of lick bouts per session, and average length of lick bouts per session.

The average number of licks per 30 min session (Figure 2D) had a significant main effect of Condition (*F*_3,39_ = 98.604, *p* < 0.001). Post hoc tests revealed that each condition had different average numbers of licks per session. The average number of total bouts per session (Figure 2E) also differed by Condition (*F*_1.63,21.195_ = 17.149, *p* < 0.001). Post hoc tests showed that the number of bouts was equivalent in the Restricted and Sucralose conditions, which both had more bouts than the Light and Dark conditions. Dark had more bouts than Light did. Average length of lick bouts in seconds (Figure 2F) had a significant main effect of Condition (*F*_1.377,17.896_ = 29.709, *p* < 0.001). Post hoc tests showed that lick bouts were shorter in the Sucralose condition than any of the others.

Thus, by manipulating three variables—hunger state, time of day, and nutritional value of the solution—we created multiple different food intake scenarios that we could analyze to see whether each factor affected measured histamine release.

#### 3.2.2 Expression of HisLightG in the PVH and in the VMH

**FIGURE 3:**
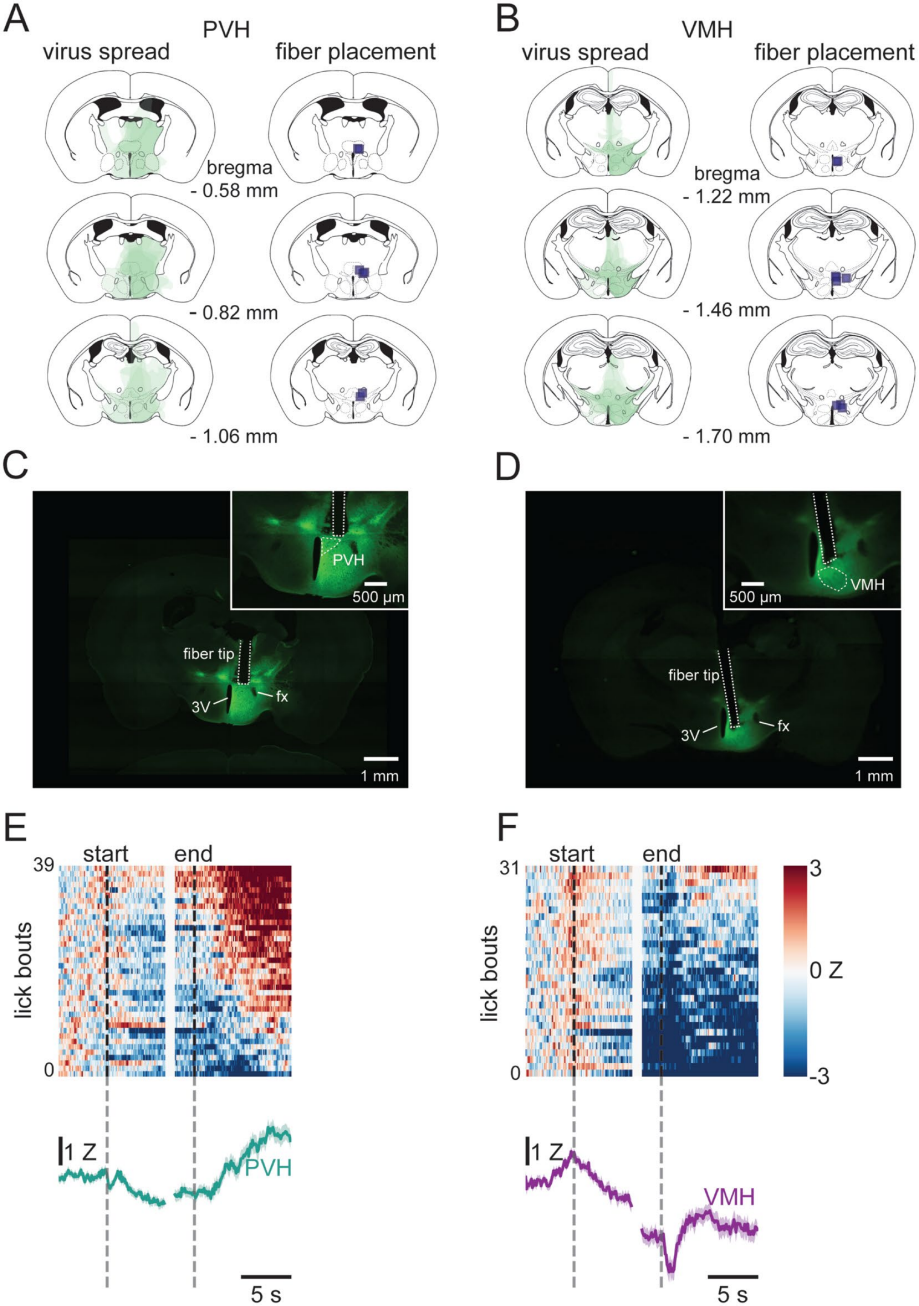
Histology and representative photometry responses in PVH and VMH. Illustration of viral spread and fiber tip location in mice included in the PVH analysis (A) and VMH analysis (B) with approximate bregma indicated. The green color shows the presence of HislightG and the blue color the fiber tip; each animal (*n* = 7 per region) is represented by a semi‐transparent shape. Images of representative animals with PVH (C) and VMH (D) injections are also shown. 3 V, third ventricle; fx, fornix. A heatmap showing the z‐scored change in fluorescence before, during, and after a lick bout for all valid lick bouts (*n* = 39) in a representative mouse with HisLightG in the PVH, as well as the trace of the average of those trials (E). The individual lick bouts (*n* = 31) and the average of those for a mouse with HisLightG in the VMH (F).

We examined viral spread and fiber placement in brains from mice in the PVH cohort (*n* = 11) and the VMH cohort (*n* = 12). In the PVH cohort 4 mice were excluded due to not having viral expression in the PVH, and in the VMH cohort 2 mice were excluded for poor virus expression and 3 for improper fiber location. This left a total of 7 mice for each region; all subsequent analyses were restricted to mice with viral expression in, and fiber tip near, the PVH (Figure 3A and Figure 3C) or VMH (Figure 3B and Figure 3D).

#### 3.2.3 Changes in Histamine Associated With Lick Bouts, in the PVH and VMH

Our primary interest was in examining how histamine may fluctuate during and around consumption. As seen above, the most lick bouts occurred during the Restricted condition, so we looked closely at mice in this condition expressing HisLightG in the PVH and in the VMH.

The z‐scored change in fluorescence associated with lick bouts showed a similar pattern across lick bouts and sessions in both the representative PVH mouse (Figure 3E) and VMH mouse (Figure 3F), and it appeared that there were some differences between the regions, especially after the end of the lick bout.

**FIGURE 4:**
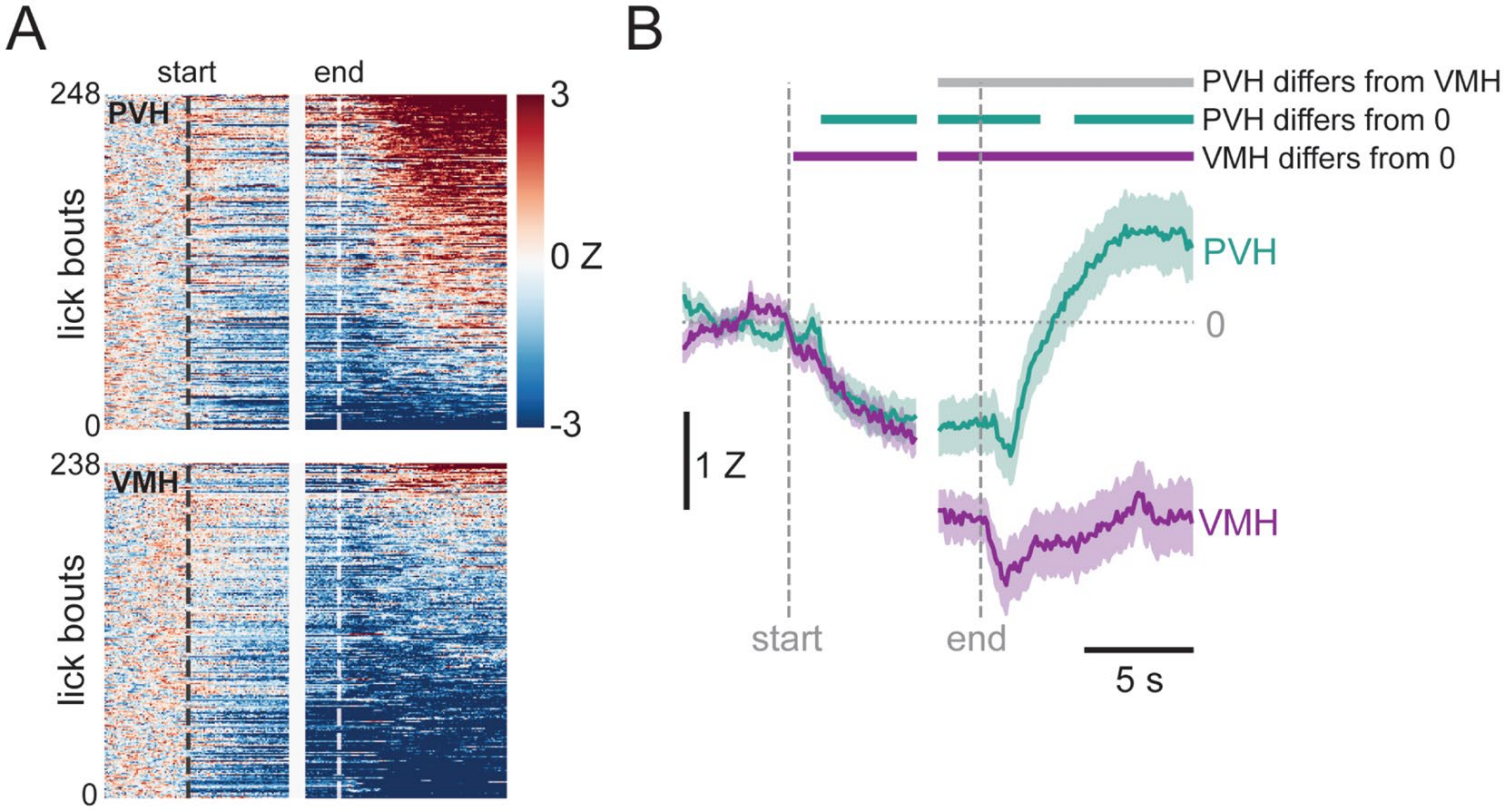
Histamine in the PVH and VMH during consumption of “Ensure” while food restricted. Each lick bout that met the criteria for photometry analysis was plotted in a heatmap, sorted based on post‐licking z‐score. This reveals an apparent difference between the PVH and the VMH (A). The difference in histamine was confirmed by waveform analysis, which showed that these regions differed from each other from the last 2 s of licking until at least 10 s after licking stopped; in the VMH histamine remained below baseline from the beginning of the lick bout until the end of the 10 s post‐lick period, whereas in the PVH histamine was reduced during licking and for the first seconds after licking stopped, at which point it rebounded to above baseline levels (B).

To analyze these changes, we performed waveform analysis on histamine fluctuations in the PVH and VMH during the Restricted condition (Jean‐Richard‐dit‐Bressel et al. 2020). A total of 248 lick bouts (each of the 7 mice contributing between 18 and 45 bouts) from the PVH and 238 lick bouts (each of the 7 mice contributing between 19 and 43 bouts) from the VMH were included (Figure 4A). These lick bouts were used to construct a bootstrapped mean and 95% confidence interval for each region. In the PVH, histamine was reduced below zero 1.5 s after licking started and remained below zero for 2.8 s after licking stopped. Between 4.4 s and 10 s after licking stopped, histamine in the PVH rebounded to above zero. In the VMH, however, histamine fell below zero 0.2 s after licking started and remained suppressed throughout the lick bout and for at least 10 s after licking ceased. Comparing the confidence intervals for the histamine signal in the PVH and in the VMH revealed a significant difference (i.e., no overlap between the two regions) in the last 2 s of licking and throughout the 10 s of post‐licking examined; during this time, histamine was lower in the VMH than in the PVH (Figure 4B).

In addition to the waveform analysis, we conducted a more traditional area‐under‐the‐curve (AUC) analysis with epochs that we designated based on the behavior (i.e., the beginning and end of a lick bout, and the 10 s following a lick bout divided into two equal 5 s bins). Overall, the conclusions drawn from the AUC analysis were aligned with those from with the waveform analysis (Figure S1). As such, both regions responded with a similar decrease in histamine during the first 6 s of licking (“early licking”) but by the end of the lick bout although both regions had a sustained decrease, histamine was lower in VMH than PVH (“late licking”). After licking stopped, histamine rapidly rebounded in PVH but remained below baseline for at least 10 s in VMH (“early post” and “late post”).

#### 3.2.4 Changes in Histamine Across Time of Day, Hunger State, and Caloric Content

**FIGURE 5:**
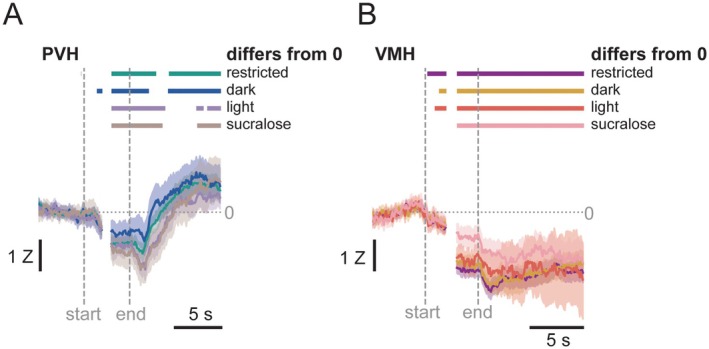
Histamine release across different conditions. In the PVH (A), histamine traces show no major differences between Dark and Restricted conditions, but a slower rebound in the post‐lick period in the Light and Sucralose. In the VMH (B), there were no differences in histamine in the Restricted, Dark, and Light conditions, but no reduction in histamine during early licking in the Sucralose condition.

To see if histamine was sensitive to time of day, hunger state, or the caloric content of the solution, we compared the histamine response between conditions in the PVH (Figure 5A) and in the VMH (Figure 5B). Total lick bouts included in this analysis and the number of bouts from each mouse can be found in Table 2. In the PVH, we saw little difference in histamine in the Restricted and Dark conditions. In the Light and Sucralose condition, histamine took longer to rebound to above zero after licking stopped. In the VMH, histamine was suppressed to below zero in the Restricted, Dark, and Light conditions within a second of licking and remaining low for the full 10 s after licking stopped; in the Sucralose condition histamine did not fall below zero until the last 2 s of the lick bout.

**TABLE 2:** Number of lick bouts contributing to the photometry analysis.

Region	Condition	Total lick bouts	Bouts per mouse
PVH	Restricted	248	18–54
	Dark	107	5–23
	Light	104	9–22
	Sucralose	85	4–22
VMH	Restricted	270	19–54
	Dark	126	10–23
	Light	77	2–22
	Sucralose	74	8–16

*Note:* Number of lick bouts > 4 s that were used to analyze changes in histamine during different conditions. Total lick bouts included, as well as number from each of the mice, are displayed.

### 3.3 Behavioral Manipulation of Histamine Levels

**FIGURE 6:**
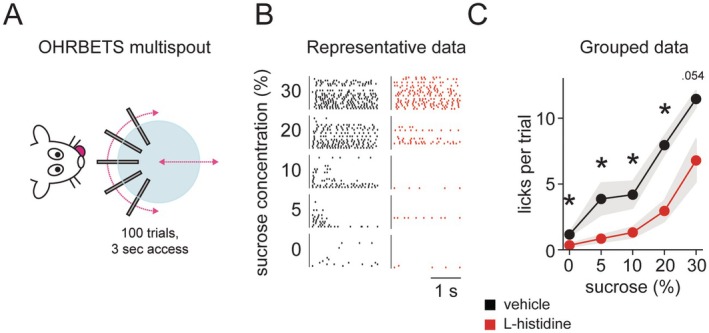
Increasing histamine suppresses sucrose intake. Mice were trained to lick in an OHRBETS multispout apparatus (A). Raster plots from representative mice show less licking in the mouse given a systemic injection of the histamine precursor, L‐histidine (B), and this was also evident in the grouped data where total licks were reduced when mice were given L‐histidine. Main effects of both sucrose concentration and drug administration were observed with planned comparisons showing that licks per trial were lower to at all sucrose concentrations when mice were administered L‐histidine (C). Plotted data are mean with shaded area as SEM. *p < 0.05.

We examined licking behavior to different concentrations of sucrose in the OHRBETS multispout in mice given injections of the histamine precursor, L‐histidine, or vehicle (Figure 6A). Raster plots with data from representative mice are shown in Figure 6B. These data indicate that increased licking was observed for higher concentrations of sucrose and that L‐histidine suppressed this licking. Analysis of grouped data by repeated‐measures ANOVA found a main effect of injection (saline, mean 572.38 +/− SEM 64.22 vs. L‐histidine, 244.88 +/− 58.09; F_1,7_ = 23.024 *p* = 0.002), such that mice with L‐histidine injections licked less overall than when they were given vehicle injections. There was also a main effect of sucrose concentration (F_4,28_ = 52.342, *p* < 0.001), with mice licking more to higher concentrations of sucrose. The interaction between sucrose concentration and injection type was not significant (Figure 6C; *F*_1.297,9.081_ = 2.051, *p* = 0.187). We performed planned comparisons between injection types at each sucrose concentration and found that mice with L‐histidine injections licked less than control mice at 0% (*t*_7_ = −3.066, *p* = 0.018), 5% (*t*_7_ = −2.553, *p* = 0.03), 10% (*t*_7_ = −2.786, *p* = 0.027), 20% (*t*_7_ = −4.614, p = 0.002), and a trend towards less at 30% sucrose (*t*_7_ = −2.312, *p* = 0.054).

## 4 Discussion

Here, we show for the first time that a novel fluorescent histamine sensor, HisLightG, is able to detect rapid fluctuations in histamine signaling while animals engage in ingestive behavior. Although previous studies have used fiber photometry to measure histamine in vivo (Dong et al. 2023; Lin et al. 2023; Xu et al. 2022), these studies used a different sensor (GRAB‐HA1m or GRAB‐HA1h). The availability of multiple sensors to detect a single neurotransmitter is useful, as differences between individual sensor properties (e.g., brightness, affinity, and ligand specificity) can make a particular sensor more suitable for a given experiment; this has been discussed in detail for dopamine sensors (Labouesse et al. 2020).

Our in vitro experiments clearly show that HisLightG responds to histamine. The response to histamine compared to a variety of other neurotransmitters shows a high specificity to histamine. Interestingly, acetylcholine elicited a small response when applied at a high concentration. However, this fluorescent response was an order of magnitude smaller than that of histamine, and indeed so small that it is extremely unlikely to cause any detectable signals in vivo. In light of this, we have a high degree of confidence that our photometry experiments were reliably measuring histamine release and not acetylcholine. We also show here that one of the two previously available sensors (specifically GRAB‐HA1h) exhibits a large negative response to acetylcholine, applied at the same concentration as mentioned above for HisLightG. Such a negative response could be cause for concern when using this tool in vivo. Thus, the combination of the intermediate brightness, high affinity, and high ligand specificity of HisLightG makes it a better option than either GRAB‐HA1h or GRAB‐HA1m for monitoring low‐level histamine release in vivo.

Past studies that have prevented histamine from exerting its normal function in particular brain areas have identified the VMH and PVH as the regions responsible for histamine reducing food intake (Ookuma et al. 1989, 1993; Sakata et al. 1988, 1990, 1991). Here, we examined the dynamics of histamine in these areas during eating behavior and found both similarities and differences in the histaminergic responses to consumption. In both regions, in food‐restricted mice, histamine decreased as licking commenced. It remained below baseline when licking stopped. In the PVH, however, histamine rebounded to above baseline levels following the end of licking, while in the VMH it remained suppressed. The functional implications of this difference require further investigation. A previous microdialysis experiment demonstrated a brief rise in medial hypothalamic histamine after food‐deprived rats were given 15 min to eat (Itoh et al. 1991). This increase in histamine was present on a timescale of minutes, indicating a modestly prolonged response of histamine to food. We show that histamine is also altered on a timescale of seconds during consumption of nutritive solutions when hungry, with changes closely linked to individual lick bouts. Interestingly, the study by Itoh et al. (1991) found an increase in medial hypothalamus histamine after eating, while we found regional differences such that in the PVH histamine increased after an eating bout, but the opposite was true in the VMH.

When we examined histamine release in each region during different conditions, we again found some regional differences. In the PVH, histamine was suppressed during the lick bout in all conditions. After the lick bout, histamine rebounded in the Restricted and Dark conditions. In the Light and Sucralose conditions, there was also a rebound but it was slower to occur. This leaves open the possibility that hunger state and caloric content of the solution influence the rebound of histamine in the PVH. Mice in the Restricted condition were hungry because of the food restriction, and in the Dark condition were presumably hungry simply from it being the time of day they normally eat. In the Light condition, the mice were likely less hungry because during the light phase they are normally sleeping, not eating, and so when these mice licked for Ensure, histamine release was different than when these same mice consumed Ensure in a different hunger state. In the Sucralose condition, the mice were tested in the dark phase and therefore equally hungry as when tested in the Dark phase, but the substance they were given to lick contained no calories. Again, this appears to have influenced histamine release in the PVH. In the VMH, in contrast, histamine remained suppressed from the start of licking until at least 10 s after licking stopped in the Restricted, Dark, and Light conditions. In the Sucralose condition, histamine showed no change from baseline during the first 2 s of licking, but was then suppressed equally as in the other conditions.

Caloric content is one possible reason why histamine responses were different in the Sucralose condition compared to the conditions in which mice were drinking Ensure, but it is also plausible that the difference was instead taste‐driven. Microdialysis experiments measuring histamine in the anterior hypothalamus found that histamine levels respond to the nature of the ingested substance. Specifically, perfusates from anterior hypothalamus of food deprived rats receiving intraoral infusions of various tastants showed increased histamine after sodium chloride, hydrochloric acid, and quinine, but a decreased histamine response after saccharin or sucrose (Treesukosol et al. 2005). Interestingly, when saccharin or sucrose was paired with lithium chloride to produce a conditioned taste aversion, these tastes produced an increase in histamine (Treesukosol et al. 2005). The authors conclude that histamine release may reflect palatability of the ingested substance, with decreases indicative of high palatability. Both of our test solutions—“Ensure” and sucralose—are generally considered palatable, and this is consistent with the reduced histamine seen during licking. Whether this is related to the sustained histamine suppression in the VMH after licking stops, and why the same solutions caused an increase after licking in the PVH, requires further study.

A fall in VMH histamine could reflect the palatability of the solution, but it is also likely that it is related to the role of histamine in suppressing food intake. Higher levels of histamine are associated with decreased food intake, so the reduction in histamine during eating could be promoting continued consumption, both within that lick bout and increasing the likelihood of subsequent lick bouts occurring due to the continued suppression. The fact that histamine in the PVH rebounds to above baseline after a lick bout—rather than remaining suppressed as in the VMH—could mean that these regions have distinct roles in appetite regulation. For example, it could be that histamine in the VMH is more related to within‐meal decisions (e.g., whether to continue an eating bout or end a meal), whereas histamine in the PVH is more related to long‐term energy balance. This is highly speculative and requires further testing. If this were true, we might expect manipulations of histamine in the VMH to affect the number of lick bouts, whereas those same manipulations in the PVH would have less of an effect on number and size of individual bouts, but might affect intake over a longer period of time.

To demonstrate a role for increased histamine levels in suppressing feeding, we performed a test in a recently developed multispout apparatus (Gordon‐Fennell et al. 2023) in which it was possible to test licking across several different concentrations of sucrose. In this test, we found that increasing histamine levels by injecting the histamine precursor, L‐histidine, suppressed licking. Thus, this links increased histamine to reduced licking and potentially satiety mechanisms, although it is important to note that in this experiment, L‐histidine was administered i.p., and therefore we cannot rule out systemic effects. Nonetheless, i.p. L‐histidine is known to increase brain levels of histamine (Itoh et al. 1984; Oishi et al. 1989). Future experiments using this method can determine whether antagonizing specific histamine receptors in specific brain regions is able to block this suppressive effect of L‐histidine on licking behavior.

In summary, here we show that the fluorescent histamine sensor, HisLightG, can be used to detect rapid fluctuations in histamine release associated with eating bouts and that these patterns of release show some regional heterogeneity and sensitivity to the caloric content of the ingested substance.

## Author Contributions

**K. L. Volcko:** conceptualization, investigation, formal analysis, writing – original draft. **A. Gresch:** investigation. **B. Benowitz:** investigation. **H. Taghipourbibalan:** investigation. **M. Visser:** investigation. **V. Rohner:** investigation. **G. D. Stuber:** conceptualization, funding acquisition. **A. G. Gordon‐Fennell:** conceptualization, investigation. **T. Patriarchi:** conceptualization, funding acquisition. **J. E. McCutcheon:** conceptualization, investigation, formal analysis, writing – original draft, funding acquisition.

## Conflicts of Interest

The authors declare no conflicts of interest.

## Peer Review

The peer review history for this article is available at https://www.webofscience.com/api/gateway/wos/peer‐review/10.1111/jnc.70142.

## Supporting information


Appendix S1.

## Data Availability

Raw data files are available at the following link: http://doi.org/10.5281/zenodo.13981427. Data were extracted using custom Python scripts available at https://github.com/mccutcheonlab/hislightg. A preprint of this article was posted on March 7 at https://www.biorxiv.org/content/10.1101/2024.11.07.622425v3.
