# Supplementary material for: Histamine Dynamics During Ingestive Behavior Measured by the Novel Biosensor HisLightG
Source: J Neurochem. 2025 Jul 3;169(7):e70142. doi: 10.1111/jnc.70142 (PMC12232117; doi:10.1111/jnc.70142)
Supplement: Supplementary file 1 — Appendix S1. [file JNC-169-0-s001.pdf]

Supplemental materials

Histamine dynamics during ingestive behavior measured by the novel biosensor HisLightG

Authors: Volcko KL[1], Gresch A[2], Benowitz B[3], Taghipourbibalan H[1], Visser M[1], Rohner V[2], Stuber GD[3], Gordon-Fennell AG[3], Patriarchi T[2,4], McCutcheon JE[1]

Supplemental Methods

Sample size calculations and estimates of achieved power

For in vitro studies, we did not calculate sample sizes but used well-characterized methods for determining sensitivity and specificity of fluorescent sensors in in vitro preparations. Using G*Power to determine achieved power based on an effect size of 9.95, confirms that we had power (1 – β error probability) = 1.0.

For in vivo photometry experiments, it was difficult to calculate the sample size a priori as the sensor had never been used before so an accurate effect size estimate was not available. Therefore, we based the number of animals on previous studies of this kind taking into account expected attrition and experimental logistics as well as a priori sample size calculations for similar experiments. For the photometry analysis, waveform analysis was used (Jean-Richard-dit-Bressel et al., 2020) to avoid having to pre-specify AUC windows. Thus, changes in responses were identified in an unbiased way using boot-strapped estimates for confidence intervals that were derived from trial-to-trial variability in the signal. In addition, we used a consecutive threshold equal to the bandwidth (i.e., six consecutive samples needed to be significant to accept that epoch as being significantly different). The effect of these analysis choices on familywise Type 1 error rates and Type 2 error rates are explored fully in Jean-Richard-dit-Bressel et al. (2020) and based on calculations this should result in high power (e.g., >0.8) for our analyses.

For the accompanying behavioral component in which data from both target sites were pooled and mouse was the unit of statistic, we had a sample size of 14. We used G*Power to determine power based on the following effect sizes from our repeated-measures, within-subject ANOVA: number of licks: effect size, 2.76 and power = 1.0; number of lick bouts: effect size, 1.24 and power = 1.0; bout length: effect size, 1.513 and power = 1.0.

For multispout experiments, the number of animals was based on previous studies using within-subject comparisons of sucrose concentration and experimental manipulations (e.g., drug responses) (Gordon-Fennell et al., 2023). Effect sizes in this type of experiment are typically high due to the within-subject nature of the testing (i.e., multiple trials of each different concentration per session). Accordingly, we used G*Power to calculate effect sizes (f values) for the repeated measures ANOVA to be as follows: 2.73 (sucrose concentration effect), 1.81 (injection effect), and 0.54 (interaction) all of which yielded achieved power of >0.99.

Supplemental Figures

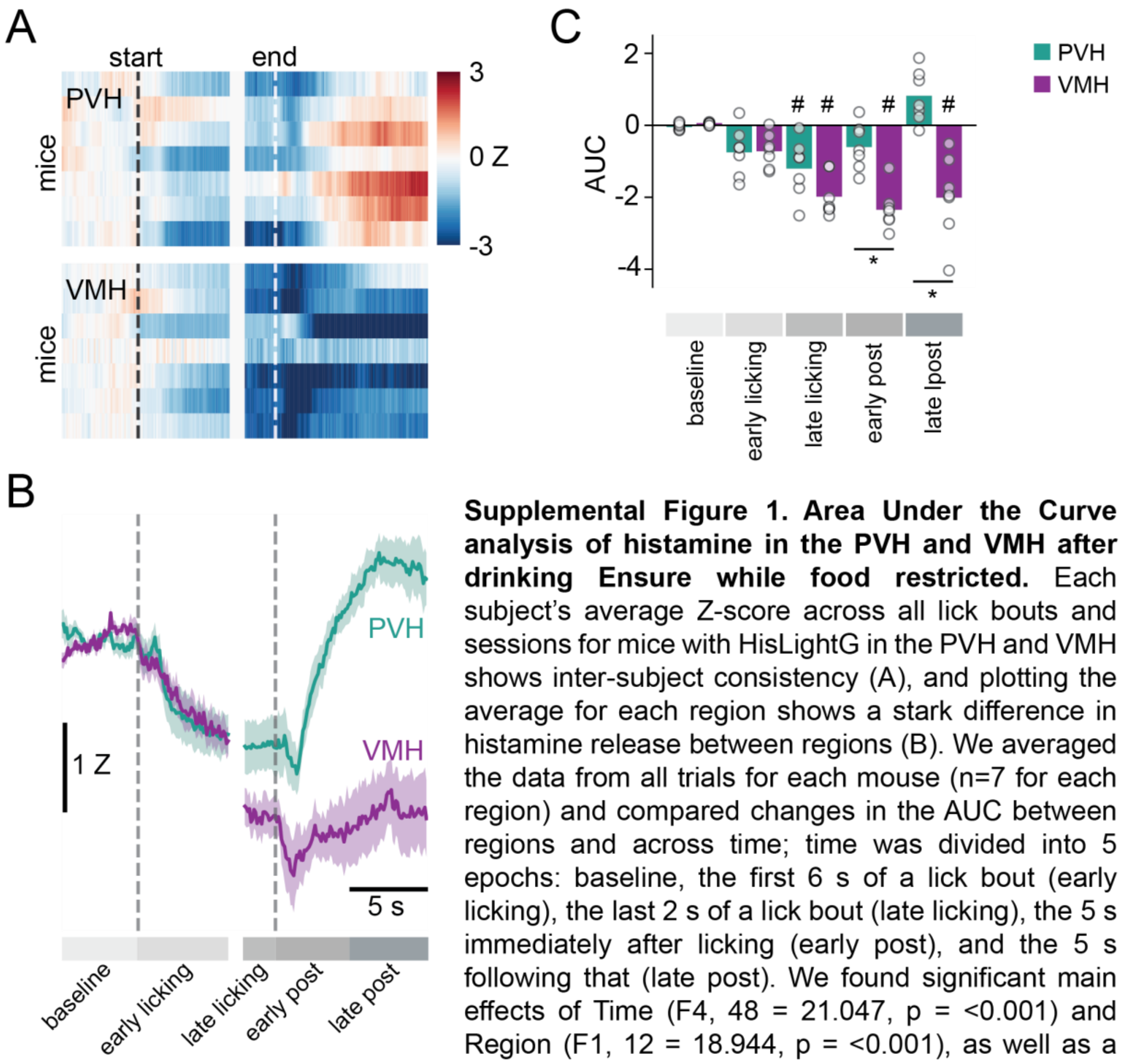

Supplemental Figure 1. Area Under the Curve analysis of histamine in the PVH and VMH after drinking Ensure while food restricted. Each subject's average Z-score across all lick bouts and sessions for mice with HisLightG in the PVH and VMH shows inter-subject consistency (A), and plotting the average for each region shows a stark difference in histamine release between regions (B). We averaged the data from all trials for each mouse (n=7 for each region) and compared changes in the AUC between regions and across time; time was divided into 5 epochs: baseline, the first 6 s of a lick bout (early licking), the last 2 s of a lick bout (late licking), the 5 s immediately after licking (early post), and the 5 s following that (late post). We found significant main effects of Time ($F4, 48 = 21.047$, $p = <0.001$) and Region ($F1, 12 = 18.944$, $p = <0.001$), as well as a significant interaction between the two ($F4, 48 = 18.518$, $p = <0.001$). Holm-corrected post hoc tests revealed that in the PVH, only the late licking epoch had a lower AUC than the baseline epoch, while in the VMH, the late licking, early post, and late post epochs all had lower AUCs than baseline. Furthermore, in both post-licking epochs, the AUC of PVH and VMH differed from one another (C). Bars are means and circles individual data points, # $p < 0.05$ from baseline for that region, * $p < 0.05$ between regions for that epoch.

References

Gordon-Fennell, A., Barbakh, J. M., Utley, M. T., Singh, S., Bazzino, P., Gowrishankar, R., Bruchas, M. R., Roitman, M. F., & Stuber, G. D. (2023). An open-source platform for head-fixed operant and consummatory behavior. *eLife*, *12*, e86183. https://doi.org/10.7554/eLife.86183

Jean-Richard-dit-Bressel, P., Clifford, C. W. G., & McNally, G. P. (2020). Analyzing Event-Related Transients: Confidence Intervals, Permutation Tests, and Consecutive Thresholds. *Frontiers in Molecular Neuroscience*, *13*. https://doi.org/10.3389/fnmol.2020.00014
